# The Potential Role of m6A RNA Methylation in the Aging Process and Aging-Associated Diseases

**DOI:** 10.3389/fgene.2022.869950

**Published:** 2022-04-20

**Authors:** Jin Sun, Bokai Cheng, Yongkang Su, Man Li, Shouyuan Ma, Yan Zhang, Anhang Zhang, Shuang Cai, Qiligeer Bao, Shuxia Wang, Ping Zhu

**Affiliations:** ^1^ Department of Geriatrics, The Second Medical Center and National Clinical Research Center for Geriatric Diseases, Chinese PLA General Hospital, Beijing, China; ^2^ Medical School of Chinese PLA, Beijing, China; ^3^ Department of Geriatric Cardiology, The Second Medical Center, Chinese PLA General Hospital, Beijing, China; ^4^ Department of Outpatient, The First Medical Center, Chinese PLA General Hospital, Beijing, China

**Keywords:** N6-methyladenosine, aging, aging-related disease, epigenetics, RNA methylation

## Abstract

N6-methyladenosine (m^6^A) is the most common and conserved internal eukaryotic mRNA modification. m^6^A modification is a dynamic and reversible post-transcriptional regulatory modification, initiated by methylase and removed by RNA demethylase. m^6^A-binding proteins recognise the m^6^A modification to regulate gene expression. Recent studies have shown that altered m^6^A levels and abnormal regulator expression are crucial in the ageing process and the occurrence of age-related diseases. In this review, we summarise some key findings in the field of m^6^A modification in the ageing process and age-related diseases, including cell senescence, autophagy, inflammation, oxidative stress, DNA damage, tumours, neurodegenerative diseases, diabetes, and cardiovascular diseases (CVDs). We focused on the biological function and potential molecular mechanisms of m^6^A RNA methylation in ageing and age-related disease progression. We believe that m^6^A modification may provide a new target for anti-ageing therapies.

## 1 Introduction

Ageing is a process of molecular and cellular damage accumulating over time, leading to a progressive decline in physical and mental capacity and an increased risk of disease and death (Borghesan et al., 2020). At present, changes in molecular and cellular ageing processes are believed to be the basis of age-related diseases, including cell senescence, autophagy, inflammation, oxidative stress, DNA damage, telomere depletion, protease inactivation, and epigenetic disorders (Ungvari et al., 2020). Ageing is the greatest risk factor for most chronic diseases, leading to morbidity and mortality (Kennedy et al., 2014). Presently, the field of ageing has focused on understanding the molecular mechanisms that regulate the ageing process and identifying biomarkers that could help to predict age-related processes. New therapeutic targets mainly focus on improving the health of the elderly population.

Epigenetics regulate gene and non-coding RNA expression without altering primary DNA sequences through many mechanisms, such as DNA methylation, histone modification, and nucleosome localisation (Portela and Esteller, 2010). Epigenetic imprinting persists during development and can be passed on to the offspring (Fraga et al., 2005; Kaminsky et al., 2009). Known epigenetic mechanisms include DNA methylation, histone modification, chromatin remodelling, and RNA methylation (Wang and Chang, 2018). At present, it is believed that during the ageing process, a decrease in histone synthesis and a change in chromatin structure leads to a general loss of structural heterochromatin (Lee et al., 2020). Histone variants have also been observed in ageing organisms, which have different primary sequences and properties compared to typical histones, thus changing the gene transcription program (Henikoff and Smith, 2015). In addition, the ageing process involves DNA methylation changes (Day et al., 2013; Horvath, 2015; Unnikrishnan et al., 2019), ATP-dependent chromatin remodelling (Clapier et al., 2017), histone modifications (including methylation, acetylation, ubiquitination) (Lawrence et al., 2016), and miRNA changes (Huan et al., 2018).

As one of the most common post-transcriptional modifications in eukaryotic mRNA, N6-methyladenosine (m^6^A) adds a methyl group to the nitrogen-containing base at the sixth position of the adenine residue of RNA. It was first found in the eukaryotic mRNA of Novikov hepatoma cells and mouse L cells (Desrosiers et al., 1974; Schäfer, 1982). m^6^A modification has a conservative identification motif, RRACH (R = G/A, H = A/C/U) (Csepany et al., 1990). The evolutionary conservatism and dynamic reversibility of its modification make it unique for gene expression regulation. m^6^A RNA methylation has become a key regulator of various post-transcriptional gene regulation processes and acts as a translation initiation mechanism in protein synthesis (Karthiya and Khandelia, 2020). In addition, numerous reports have indicated that m^6^A modification may cause important changes in the ageing process and affect the occurrence and development of many age-related diseases. In this review, we focused on m^6^A RNA methylation mechanisms related to the ageing process and emphasised their significance in age-related diseases. We believe that m^6^A RNA methylation is a potential target for treating age-related diseases.

## 2 Overview of N6-Methyladenosine Modification

**FIGURE 1:**
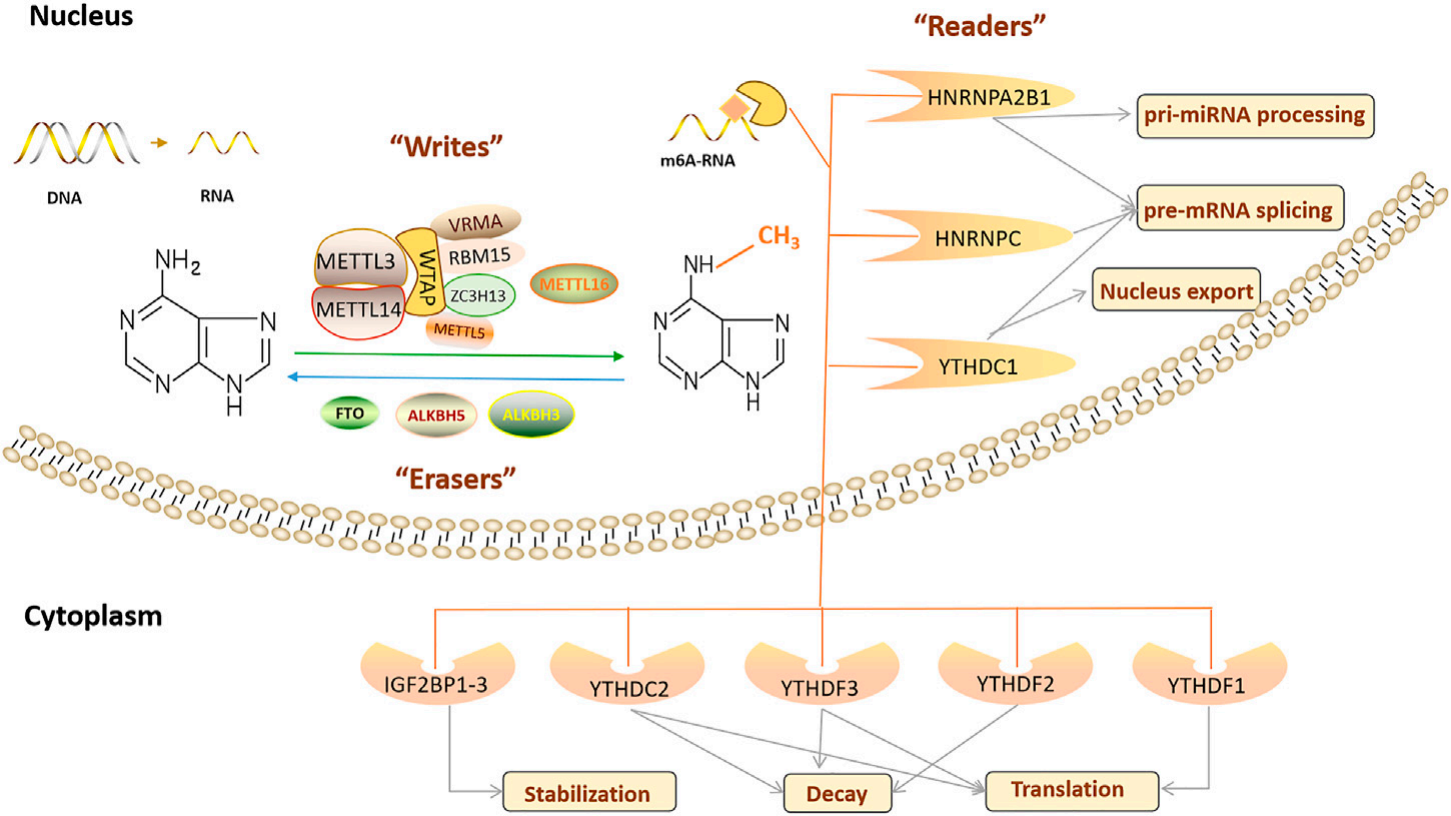
Regulation of the m6A modification and the function of the m6A regulators.

RNA modification is a post-transcriptional process that regulates gene expression by binding to proteins without involving the RNA sequence. More than 160 types of RNA modifications, ubiquitous in both coding and non-coding RNA, have been identified. First discovered in 1974, m^6^A modification refers to the methylation of the sixth nitrogen atom of adenylate. It is considered the most abundant internal modification in eukaryotic mRNA (Desrosiers et al., 1974). With recent improvements in detection techniques, such as high-throughput sequencing, the study of m^6^A RNA methylation is booming. Presently, it has been reported that there are three m^6^A residues per average mRNA transcript in mammalian cells (Dominissini et al., 2012). In addition to mRNA, m^6^A RNA methylation covers almost all types of RNA, including transfer RNAs (tRNAs), ribosomal RNAs (rRNAs), cyclic RNAs (circRNAs), microRNAs, and small nucleolar RNA (snoRNA) (Sergiev et al., 2016).

m^6^A RNA methylation is a dynamic and reversible RNA modification, and its function is determined by three types of enzymes: RNA methyltransferase, RNA demethylase, and m^6^A-binding proteins (Figure 1) (Fu et al., 2014). m^6^A modification is crucial in regulating gene expression, splicing, RNA editing, RNA stability, controlling mRNA lifespan and degradation, and mediating ring RNA translation (Zhao et al., 2017). In addition, m^6^A modification is related to many physiological processes, pathological processes, and human diseases, including the circadian rhythm (Zhong et al., 2018), reproductive system development (Hongay and Orr-Weaver, 2011; Hsu et al., 2017; Ivanova et al., 2017; Kasowitz et al., 2018), haematopoietic system development (Wang et al., 2014a; Zhang et al., 2017), nervous system development and degeneration (Hess et al., 2013; Lence et al., 2016; Li et al., 2017a; Yen and Chen, 2021), cardiovascular diseases (CVDs) (Chen et al., 2021a), nutritional and metabolic diseases (Wu et al., 2020a), and tumorigenesis (Wang et al., 2020a; Zhou et al., 2020).

### 2.1 RNA Methyltransferases

RNA methyltransferases, including RNA methyltransferase-like protein 3 (METTL3) (Bokar et al., 1997), RNA methyltransferase-like protein 14 (METTL14) (Liu et al., 2014), Wilms’ tumour 1-associating protein (WTAP) (Agarwala et al., 2012), RNA-binding motif protein 15 (RBM15) and its analogue RBM15B (Patil et al., 2016), Vir-like m^6^A RNA methyltransferase associated protein (VIRMA)/KIAA1429 (Schwartz et al., 2014), Zinc finger CCCH domain-containing protein 13 (ZC3H13) (Wen et al., 2018), RNA methyltransferase-like protein 16 (METTL16) (Pendleton et al., 2017), and RNA methyltransferase-like protein 5 (METTL5) (van Tran et al., 2019; Richard et al., 2019), mediate m^6^A modification, are mainly located in nuclear speckles, and are called “m^6^A writers.” Among these, METTL3 was the first key RNA methyltransferase and core RNA methyltransferase subunit of m^6^A methylation. It is critical in the occurrence of m^6^A modifications and participates in various physiological processes (Bokar et al., 1997). Abnormal METTL3 expression changes m^6^A RNA methylation levels. As the structural support for METTL3, METTL14 is co-located in the nucleus in a 1:1 ratio and forms a stable RNA methyltransferase complex responsible for m^6^A modification (Liu et al., 2014). WTAP in the RNA methyltransferase complex is primarily used as a connecting protein between METTL3 and METTL14. WTAP lacks a conserved catalytic methylation domain and cannot catalyse m^6^A modification, but its deletion significantly affects m^6^A modification levels and physiological processes, such as embryonic differentiation (Ping et al., 2014). METTL3/METTL14/WTAP is considered to be the core RNA methyltransferase component, and in recent years, some studies have reported new RNA methyltransferase complex components, such as RBM15/15B, which assists in the binding of METTL3 and WTAP, and its deletion leads to damage to X-inactive specific transcript (XIST)-mediated gene silencing on the X chromosome (Knuckles et al., 2018). ZC3H13 (Wen et al., 2018), VIRMA (Yue et al., 2018), and other proteins also participate in m^6^A RNA methylation as cofactors of the m^6^A RNA methyltransferase complex. In addition, Warda et al. (2017) reported on an independent m^6^A writer, METTL16, finding that its binding site does not overlap with the METTL3/METTL14 methylation complex, and it regulates the stability and splicing of mRNA by catalysing m^6^A modification in snoRNAs, U6 small nuclear RNAs (snRNAs), and other long non-coding RNAs (lncRNAs). There are continuous reports of new RNA methyltransferases, such as METTL5, the enzyme responsible for 18S rRNA m^6^A modification, and ZCCHC4, a confirmed 28S rRNA m^6^A modification enzyme (van Tran et al., 2019; Richard et al., 2019). Some studies reported that WTAP interacts with many proteins and lncRNAs, of which more than 100 may bind to METTL3 or METTL14 (Schöller et al., 2018). Therefore, “writer” may include the reported proteins and other components that need further exploration.

### 2.2 RNA Demethylases

RNA demethylases, including fat mass and obesity-related proteins (FTO) (Jia et al., 2011), AlkB homologue 5 (ALKBH5) (Huang et al., 2020a), and AlkB homologue 3 (ALKBH3) (Ueda et al., 2017; Sun et al., 2019), can remove the m^6^A modification. They are called “m^6^A erasers” and are located in nuclear spots with RNA methyltransferase. In 2011, FTO was identified as the first m^6^A RNA demethylase, verifying that m^6^A RNA methylation is a dynamic and reversible RNA modification. FTO-mediated m^6^A demethylation acts in various biological processes, inhibiting peroxisome proliferator-activated receptor (PPARβ/δ) and AMP-activated protein kinase (AMPK) pathways, disrupting skeletal muscle lipid utilisation, inhibiting macrophage lipid influx by downregulating PPARγ protein expression, and accelerating cholesterol outflow *via* AMPK phosphorylation. Thus, foam cell formation and atherosclerosis development were inhibited (Yang et al., 2022). FTO regulates the alternative splicing of RUNT-related transcription factor 1 (RUNX1) through m^6^A modifications (Zhao et al., 2014), whereas FTO regulates fat formation and deposition by altering the expression of PPARγ (Lee et al., 2011) and angiopoietin-like 4 (ANGPTL4) (Wang et al., 2015a). In addition, FTO is widely involved in regulating the cell cycle (Li et al., 2019a), tumour growth (Li et al., 2019b), proliferation and migration (Tang et al., 2019), stem cell maintenance (Su et al., 2020) and other biological processes.

ALKBH5 is the second m^6^A RNA demethylase and is expressed in most tissues, especially the testes (Aik et al., 2014). ALKBH5 inactivation increases m^6^A RNA methylation levels, leading to male-mouse infertility (Tang et al., 2018a). In addition, ALKBH3 has recently been considered a new m^6^A RNA demethylase that preferentially catalyses m^6^A demethylation in tRNA (Ueda et al., 2017; Woo and Chambers, 2019).

### 2.3 N6-Methyladenosine Binding Proteins

The “m^6^A writers” and “m^6^A erasers” determine whether RNA is methylated, but m^6^A-binding proteins (“m^6^A readers”) determine the final biological function of m^6^A modification. “m^6^A readers” recognise and bind to an m^6^A modified transcript, then regulate mRNA stability (Zhao et al., 2014), mRNA splicing (Xiao et al., 2016), mRNA structure (Spitale et al., 2015), mRNA output (Roundtree et al., 2017), translation efficiency (Wang et al., 2015b) and microRNA (miRNA) biogenesis (Alarcón et al., 2015). “Readers” include proteins containing YTH domains (YTHDF1/2/3 and YTHDC1/2), heterogeneous ribonucleoproteins including heterogenous nuclear ribonucleoprotein (HNRNP) C (HNRNPC), HNRNP G (HNRNPG), and HNRNP A2B1 (HNRNPA2B1), and insulin-like growth factor 2 binding proteins (IGF2BPs), which are members of a protein family involved in regulating some aspects of ageing. Different “readers” have different cellular localisations and thus perform various biological functions. YTH domain containing 1 (YTHDC1) regulates mRNA splicing by recruiting the splicing factor serine- and arginine-rich splicing factor 3 (SRSF3) or blocking serine- and arginine-rich splicing factor 10 (SRSF10) in the nucleus (Xiao et al., 2016). In addition, it increases the output of circRNA NOP2/SUN domain family, member 2 (circNSUN2) in the cytoplasm by interacting with nuclear output factor 1 (Chen et al., 2019a). HNRNPA2B1 and HNRNPC are also located in the nucleus. HNRNPA2B1 regulates RNA splicing and promotes miRNA maturation by recognising pri-miRNA markers and interacting with DiGeorge syndrome critical region 8 (DGCR8) (Zhao et al., 2017). HNRNPC selectively recognizes m^6^A modified transcripts to promote pre-RNA processing (Liu et al., 2015). YTHDF1/2/3, YTH domain containing 2 (YTHDC2), and IGF2BP1/2/3 are localised in the cytoplasm. YTH domain family protein 1 (YTHDF1) initiates RNA translation by interacting with translation initiation factors and ribosomes, whereas YTH domain family protein 2 (YTHDF2) selectively binds m^6^A modified transcripts and accelerates their degradation (Wang et al., 2015b). On the other hand, YTH domain family protein 3 (YTHDF3) and YTHDF1/2 play a synergistic role, not only promoting YTHDF1-mediated translation but also affecting the decline in YTHDF2-mediated m^6^A modification (Wang et al., 2014b; Shi et al., 2017). Like YTHDF3, YTHDC2 is an RNA helicase, and its helix-unwinding region contributes to RNA binding and promotes mRNA translation or degradation (Hsu et al., 2017). Other proteins located in the cytoplasm are IGF2BP1–3, which recognise and bind to m^6^A modified transcripts, thus enhancing mRNA stability (Huang et al., 2018).

## 3 N6-Methyladenosine Changes in Molecular Processes Associated With Ageing

Many studies have confirmed that m^6^A methylation regulates several physiological processes that are crucial in the ageing process. Here, we focused on the mechanisms of m^6^A RNA methylation in autophagy, inflammation, oxidative stress, DNA damage, and cell senescence (Table 1).

**TABLE 1:** The role of m^6^A modification in the fundamental processes.

The Processes related to aging	m^6^A regulator	Organism	Role in processes	Mechanism	Reference
Autophagy	MTC	Cells, Drosophila	Suppression	Promote the degradation of ATG transcripts	Tang et al. (2021)
	METTL14	Leydig Cells	Suppression	Reduce AMPK activity	Chen et al. (2021b)
	ALKBH5	Leydig Cells	Promotion	Promote the activity of AMPK	Chen et al. (2021b)
		ovarian cancer cells	Suppression	Regulation of bcl-2 expression	Zhu et al. (2019)
	FTO, YTHDF2	Cells	Promotion	Increase the expression of ULK1	Jin et al. (2018)
Inflammation	METTL3	Cells	Promotion	Regulate alternative splicing of MyD88	Feng et al. (2018)
	METTL14	Endothelial cell, mice	Promotion	Promote FOXO1 expression	Jian et al. (2020)
	ALKBH5	HK-2 cells	Promotion	Up-regulate MALAT1 expression by demethylation	Zhu and Lu, (2020)
	FTO	Cells	Promotion	Promote M1 and M2 macrophage activation	Gu et al. (2020)
	RBM4, YTHDF2	Cells	Suppression	Decrease m^6^A modified STAT1 mRNA levels and inhibite the polarization of M1 macrophages	Huangfu et al. (2020)
Oxidative stress	METTL3	mRTECs	Suppression	Regulate Keap1-Nrf2 pathway	Wang et al. (2019c)
	METTL14	Cardiomyocytes, mice	Suppression	Regulate Wnt1/β-Catenin Signaling Pathway	Pang et al. (2021)
	WTAP	Cells and rat	Promotion	Regulate m^6^A modification of ATF4 mRNA	Wang et al. (2021)
	FTO	Cell, human samples	Promotion	Increased the translation efficiency of PGC1αmRNA	Zhuang et al. (2019)
	YTHDF1/3	Cells	Promotion	Promote stress granule formation	Fu and Zhuang, (2020)
DNA damage	METTL3, YTHDC1	Cells	Suppression	Modulates accumulation of DNA-RNA hybrids at DSBs sites and recruit RAD51 and BRCA1	Zhang et al. (2020c)
	METTL3/14, YTHDC1	Cells	Suppression	Active on ssDNA and lesion-containing dsDNA	Yu et al. (2021)
	YTHDF1	Cells, mice	Suppression	Upregulates HR-related factors RAD51 and BRCA1	Sun et al. (2022)
Cell senescence	METTL3	Cells	Promotion	Target NF-κB, drives the senescence-associated secretory phenotype	Liu et al. (2021)
	METTL14	Clinical Sample	Promotion	Participates in the TNF-α-induced m^6^A modification of miR-34a-5p to promote cell senescence	Zhu et al. (2021b)
	FTO	Granulosa cells	Suppression	Regulates the expression of FOS	Jiang et al. (2021)
	METTL3, IGF2BP2	hMSC	Suppression	Stabilizate of the MIS12 transcript	Wu et al. (2020b)

### 3.1 N6-Methyladenosine and Autophagy

Autophagy is a highly conserved intracellular clearance mechanism regulated by various proteins and is important for maintaining homeostasis in the internal environment. The mammalian target of rapamycin (mTOR) is a key factor in autophagy regulation. Protein kinase B (AKT) and mitogen-activated protein kinase (MAPK) signalling pathways activate mTOR to inhibit autophagy, whereas AMPK and p53 pathways negatively regulate mTOR to promote autophagy (Alers et al., 2012). After mTOR inactivation, UNC-51-like kinase 1/2 (ULK1/2) is activated and binds to the focal adhesion kinase family interacting protein of 200 kDa (FIP200) to form a ULK1 complex with autophagy-related 13 (ATG13) proteins, promoting autophagosome formation (Codogno et al., 2011). m^6^A methylation and related regulators regulate autophagy by regulating ATG expression or by affecting autophagy-related signalling pathways. In 2018, Jin et al. first reported a positive regulatory effect of FTO on autophagy, accomplished by affecting the abundance of Unc-51 like autophagy activating kinase 1 (ULK1) (Jin et al., 2018). Another RNA demethylase, ALKBH5, has been shown to enhance autophagy by reducing m^6^A methylation in FIP200 transcripts (Li et al., 2020), suggesting a negative correlation between m^6^A modification and autophagy. A study of RNA methyltransferases further confirmed this. METTL3 upregulates methylation and triggers YTHDF1 and Forkhead box O3 (FOXO3) binding to promote the translation of FOXO mRNA. FOXO further blocks ATG gene expression to inhibit autophagy (Lin et al., 2020). A decrease in METTL14 levels increases the stability of calcium/calmodulin-dependent protein kinase 2 (CAMKK2) mRNA and activates the AMPK and ULK1 complex to initiate autophagy (Chen et al., 2021b).

Abnormal autophagy can lead to diseases, some of which may be associated with ageing. Studies have shown that autophagy decreases with age. Increasing autophagy levels can inhibit the accumulation of damaged proteins, delay the occurrence of degenerative changes, and prolong life (Rubinsztein et al., 2011; Papp et al., 2016). There is evidence that autophagy regulates some age-related diseases in lower organisms (such as *Drosophila* and *Caenorhabditis elegans*), but this hypothesis has not been confirmed in mammals. Accelerating ageing by decreasing autophagy is controversial. Nevertheless, several studies have reported that deleting autophagy proteins leads to the accumulation of misfolded proteins and abnormal mitochondria in cells, resulting in premature senescence, organ dysfunction, and eventually the development of various ageing-related diseases, such as neurodegenerative diseases, cancer, CVDs, and metabolic syndrome (Linton et al., 2015; Guo et al., 2018; Luo et al., 2020). In summary, autophagy regulation is closely related to ageing, in which m^6^A modification plays an important role. Therefore, further studies on the relationship between m^6^A modification and autophagy in ageing may provide a new method for anti-ageing research.

### 3.2 N6-Methyladenosine and Inflammation

RNA methylation is involved in inflammation. m^6^A methylation affects pathways related to metabolic reprogramming, stress response, and ageing by regulating type I interferon (IFN) mRNA stability (Rubio et al., 2018). Lipopolysaccharides (LPSs) induce inflammation. It has been found that LPS stimulation promotes METTL3 expression and biological activity in macrophages, and METTL3 overexpression alleviates lipopolysaccharide-induced inflammation through the nuclear factor-κB (NF-κB) signalling pathway, further confirming the relationship between m^6^A methylation and inflammation (Wang et al., 2019a). In addition, the interaction between m^6^A modification and inflammation is crucial for various diseases to occur. YTHDF2 deletion aggravates the inflammatory state and metastasis of human hepatocellular carcinoma cells (Hou et al., 2019). After an ischaemic stroke, FTO expression is downregulated, and m^6^A methylation is increased in the main inflammatory pathways, including interleukin (IL)-6 cytokines, tumour necrosis factor (TNF), toll-like receptor (TLR), and NF-κB signalling pathways (Chokkalla et al., 2019). It has been suggested that m^6^A may regulate secondary brain injury after cerebral ischaemia by affecting inflammation.

In summary, m^6^A methylation affects inflammation under physiological and pathological conditions. Presently, the chronic inflammatory state is considered one of the characteristics of ageing, namely “inflammatory ageing” (inflamm-ageing), which is mainly characterised by inflammatory cell infiltration and an increase in pro-inflammatory factors [TNF-α, IL-1β, IL-6, C-reactive protein (CRP), etc.] Although most current studies on the relationship between m^6^A modification and inflammation are based on specific diseases and signalling pathways, the study of epigenetic changes in inflammation potentiates the development of effective drugs with specific anti-ageing targets.

### 3.3 N6-Methyladenosine and Mitochondria: Oxidative Stress

Oxidative damage accumulates with ageing in many species and tissues. RNA modification is mobilised to activate or inhibit stress-resistant signalling pathways (Peters et al., 2021). Li et al. (2017b) found that the activities of METTL3/METLL14, p21, and senescence-related β-galactosidase (SA-βGAL) increased significantly after oxidative damage stimulated HCT116 p53^−/−^cells, indicating that METTL3/METLL14 may trigger the p53 independent effect of ageing in the oxidative damage response, which needs to be further tested. Arsenite et al. stimulated human keratinocytes to induce reactive oxygen species (ROS) production, increasing WTAP, METTL14, and total m^6^A expression levels (Zhao et al., 2019). FTO induces oxidative stress and increases ROS levels by reducing m^6^A methylation of peroxisome proliferator-activated receptor gamma coactivator-1 alpha (PGC1α) (an important regulator of mitochondrial metabolism that is also affected by the ageing process) and increasing PGC1α mRNA translation efficiency.

### 3.4 N6-Methyladenosine and DNA Damage

DNA damage refers to changes in DNA structure caused by physical or chemical stimuli in the environment. The persistence of DNA damage can lead to a prolonged DNA damage response (DDR) and induce senescence (Di Micco et al., 2021). m^6^A is critical in DNA damage and repair. It has been reported that METTL3/METTL14 and METTL16 are recruited to DNA damage sites to facilitate DNA repair and the DDR by adjusting m^6^A modifications under ultraviolet (UV) radiation stimulation (Svobodová Kovaříková et al., 2020). This repair is carried out through the nucleotide excision repair (NER) pathway because knockout of the non-homologous end junction (NHEJ) enzyme SUV391H/H2 does not affect m^6^A recruitment under UV stimulation (Svobodová Kovaříková et al., 2020).

### 3.5 N6-Methyladenosine and Cell Senescence

Cell senescence results from many processes, including telomere wear, macromolecular damage, and oncogene-activated signal transduction (Childs et al., 2015). Senescent cells widely exist in ageing and diseased tissues, secreting numerous pro-inflammatory cytokines, called the ageing-associated secretory phenotype [senescence-associated secretory phenotype (SASP)]. These cytokines regulate the tissue microenvironment and affect how nearby normal cells function. Studies have shown that senescent cells are involved in atherosclerosis (Ito et al., 2014), Alzheimer’s disease (AD) (Boccardi et al., 2015), Parkinson’s disease (PD) (Chinta et al., 2013), chronic obstructive pulmonary disease (Barnes et al., 2019), insulin resistance (Aravinthan et al., 2015), age-related chronic inflammation (Campisi and Robert, 2014), cancer (Calcinotto et al., 2019), osteoporosis (Farr and Khosla, 2019), and loss of haematopoietic stem cell function (de Haan and Lazare, 2018) in the elderly.

In 2017, Li et al. (2017b) reported a link between m^6^A methylation and cellular senescence. They found that p21 protein methylation increased with m^6^A methylation, whereas the p21 mRNA level was not affected by m^6^A, suggesting that m^6^A methylation regulates p21 translation. In another study, breast cancer cells were exposed to sublethal concentrations of ammonium trifluoride (SFN). m^6^A methylation levels decreased, the activity of SA-βGAL increased, and p53, p21, and p27 protein levels increased, but the corresponding mRNA levels remained unchanged. SFN may lead to senescence by reducing m^6^A methylation levels (Lewinska et al., 2017). Min et al. reported an m^6^A RNA modification map of human peripheral blood mononuclear cells (PBMCs) from young and old groups. They found that the total level of m^6^A modification in PBMCs of the elderly was significantly lower than that in the young PBMCs, while the expression of m^6^A modified transcripts was higher than that of unmodified transcripts (Min et al., 2018). Shafik et al. have reported dynamic changes in m^6^A RNA methylation during brain ageing. In their study, they compared the m^6^A spectra of Brodmann area 9 (BA9) in the cerebral cortex of 6-week-old and 52-week-old mice and post-mortem pubertal and elderly human brains, and the results showed that the m^6^A modification sites were significantly increased with increasing age, both in mice and humans. Functional enrichment analysis showed that differential m^6^A loci mainly occurred in the untranslated regions of genes that affect ageing-related pathways, which are related to the strong negative effect of mRNA expression (Shafik et al., 2021).

A recent study reported that METTL3 downregulation decreased m^6^A modification of human bone marrow mesenchymal stem cells (hMSC) with premature senescence, and hMSCs showed accelerated ageing after METTL3 gene knockout. The m^6^A modifications in Hutchinson-Gilford progeria (HGPS) and Werner syndrome (WS) increased with METTL3 overexpression and delayed disease progression. They identified MIS12 as the specific target of m^6^A modification deletion in the premature ageing process using RNA sequencing (RNA-seq) and m^6^A methylation RNA immunoprecipitation sequencing (MERIP-seq) analysis. m^6^A deletion accelerates hMSC ageing, while IGF2BP2 recognises and stabilizes m^6^A modified MIS12 mRNA to prevent accelerating senescence in hMSCs. Based on the above results, Wu et al. (2020b) proposed a regulatory model in which METTL3-mediated m^6^A modification improves the stability of IGF2BP2-mediated MIS12 mRNA, thus reversing the ageing phenotype of hMSCs.

Cellular senescence is an important component of the ageing process. Selective clearance of senescent cells is currently the focus of anti-senescence research. Senolytics (a mixture of dasatinib and quercetin), agents that target cellular senescence, have completed small clinical trials in patients with idiopathic fibrosis with promising efficacy and safety results (Justice et al., 2019). The results need to be validated in larger samples and populations with other age-related diseases. The link between m^6^A methylation and cellular senescence may provide novel therapeutic targets for localising senescent cells, with important clinical implications.

## 4 N6-Methyladenosine Changes in Ageing Associated Diseases/Disorders

The study of m^6^A RNA methylation and the ageing process has laid the foundation for more comprehensive and in-depth exploration into the epigenetic mechanisms of various ageing-related diseases. At present, several studies focus on the role of m^6^A RNA methylation in ageing-related pathological processes, such as cancer. Here, we summarise the latest reports on m^6^A modification and ageing-related diseases, focusing on cancer, neurodegenerative diseases, diabetes mellitus, and CVDs (Table 2).

**TABLE 2:** The functional roles of RNA m^6^A modification in various types of human disease.

Age-related disease	Organism	Role in disease	m^6^A regulator	Functional in disease	Ref
Cancer:					
Respiratory neoplasms					
Lung cancer	Clinical Samples; cells	Oncogene	METTL3; FTO; YTHDF1/2; IGF2BP1	Promote LC growth and progress; induce invasion and metastasis of NSCLC	(Lin et al., 2016; Chen et al., 2020a), (Liu et al., 2018a; Chen et al., 2018; Müller et al., 2019)
	Cells	Suppressor	ALKBH5	Inhibits tumor growth and metastasis	Jin et al. (2020)
Nasopharyngeal carcinoma	Cells	Oncogene	METTL3	Promote proliferation and invasion of NPC cells	Zheng et al. (2019)
Leukemia	Clinical Samples; cells; mice	Oncogene	METTL3; METTL14; WTAP; YTHDF1; FTO; IGF2BP1	Promote AML cells proliferation and leukemia cells self-renewal, growth and metabolism	(Bansal et al., 2014; Vu et al., 2017; Li et al., 2018a; Weng et al., 2018)
Gastroinestinal tumor					
Hepatocellular carcinoma	Clinical Samples; cells; mice	Oncogene	METTL3; METTL14; YTHDF1; KIAA1429; WTAP; YTHDF2	Induce HCC cells proliferation, migration, invasion and metastasis	(Chen et al., 2018; Cheng et al., 2019; Müller et al., 2019)
	Cells; mice	Suppressor	METTL14	Suppress tumor invasion and metastasis	Ma et al. (2017)
Gastric carcinoma	Cells, Clinical samples	Oncogene	METTL3; ALKBH5	Promote proliferation, tumor angiogenesis, invasion and metastasis of GC	(Zhang et al., 2019a; Wang et al., 2020e)
Colorectal cancer	Cells, Clinical samples, mice	Oncogene	METTL3; FTO; WTAP; YTHDC2; YTHDF1; IGF2BPs	Promote the proliferation, migration, invasion and EMT of CRC cells	(Tanabe et al., 2016; Zhang et al., 2016; Shen et al., 2018; Wu et al., 2019b; Li et al., 2019c)
	Cells, clinical samples	Suppressor	METTL3; METTL14	Suppress CRC proliferation and migration	(Deng et al., 2019; Chen et al., 2020b)
Pancreatic cancer	Cells, clinical samples	Oncogene	METTL3; YTHDF2	Promote cell proliferation, migration, and invasion	(Chen et al., 2017; Zhang et al., 2019b)
	Cells, clinical samples	Suppressor	ALKBH5; YTHDF2	Suppress cancer migration, invasion, and EMT	(Chen et al., 2017; He et al., 2018)
Urological cancers					
Bladder cancer	Cells, clinical samples, mice	Oncogene	METTL3; FTO; ALKBH5	Promote BC cells proliferation, colony formation, invasion and metastasis; inhibit cell apoptosis	(Cai et al., 2018; Wang et al., 2020f)
	Clinical samples	Suppressor	METTL14	Inhibit bladder TIC self-renewal and tumorigenesis	Gu et al. (2019)
Renal cell cancer	Cells, clinical samples, mice	Oncogene	WTAP	Enhance cell proliferation abilities	Tang et al. (2018b)
	Cells, clinical samples, mice	Suppressor	METTL3; FTO	Suppress tumor growth, proliferation, migration, invasion function and cell cycle of RCC and induce apoptosis	(Li et al., 2017d; Zhuang et al., 2019)
Prostate cancer	Cells	Oncogene	METTL3; YTHDF2	Promote tumor cells proliferation, survival, colony formation, and migration	Cai et al. (2019)
Reproductive neoplasms					
Breast cancer	Cells, clinical samples, mice	Oncogene	METTL3; FTO; ALKBH5	Promote BC cells proliferation, colony formation and metastasis; inhibit the apoptosis	(Niu et al., 2019; Wang et al., 2020f)
Ovarian cancer	Cells, clinical samples, mice	Oncogene	METTL3; ALKBH5; IGF2BP1	Promote the proliferation and invasion *in vitro* and *in vivo*	(Hua et al., 2018; Müller et al., 2019)
Cervical carcinom	Cells, clinical samples	Oncogene	FTO	Promote cell proliferation and migration; induce resistance	Zou et al. (2019)
Endometrial cancer	Cells, clinical samples, mice	Suppressor	METTL3/METTL14	Inhibit the proliferation and tumorigenicity	Liu et al. (2018b)
Skin tumors					
Melanoma	Cells, clinical samples, mice	Oncogene	FTO	Increase tumor growth	Yang et al. (2019a)
	Cells, clinical samples, mice	Suppressor	YTHDF1	Restrain cell growth and migratory ability	Jia et al. (2019)
Squamous cell carcinoma	Cells, clinical samples, mice	Oncogene	METTL3	Promote tumorigenicity	Zhou et al. (2019)
Neurodegenerative diseases:					
Alzheimer’s disease	Mice, clinical samples	Up- regulation	METTL3; IGF2BP2; RBM15B	—	(Han et al., 2020; Deng et al., 2021)
	Cells, mice, clinical samples	Down- regulation	METTL3; FTO	—	(Huang et al., 2020b; Han et al., 2020), (Zhao et al., 2021)
Parkinson’s disease	Cells	Down- regulation	HNRNPC	—	Quan et al. (2021)
Cardiovascular disease:					
Hypertension	Rat	—	—	The m^6^A methylation level reduce	Wu et al. (2019a)
Cardiac hypertrophy	Cells, mice	Up- regulation	METTL3; FTO	Promote cardiomyocyte hypertrophy both *in vitro* and *in vivo*	(Gan et al., 2013; Dorn et al., 2019), (Berulava et al., 2020)
Heart failure	Clinical samples and mice	Up- regulation	METTL3, METTL4, KIAA1429, FTO, YTHDF2	Data from MeRIP-seq	Zhang et al. (2021)
	Clinical sample, preclinical pig, mice, cells	Down- regulation	FTO	Increase m^6^A in RNA and decrease cardiomyocyte contractile function	Mathiyalagan et al. (2019)
Atherosclerosis	Cells, mice, clinical sample	Up- regulation	METTL3, METTL14, IGF2BP1	Promote cardiovascular endothelial cell proliferation and invasion; aggravates endothelial inflammation, angiogenesis and atherosclerosis	(Zhang et al., 2020b; Jian et al., 2020; Dong et al., 2021)
Diabete mellitus	Clinical sample, cells	Up- regulation	FTO, METTL3	Induce mRNA expression of FOXO1, G6PC, and DGAT2	(Yang et al., 2019b; Yang et al., 2020b)
	Cells, mice, clinical sample	Down- regulation	METTL3, METTL14	regulated functional maturation and mass expansion of neonatal β-cells	(De Jesus et al., 2019; Liu et al., 2019; Men et al., 2019; Wang et al., 2020d)

### 4.1 Cancer

In recent years, many studies on m^6^A RNA methylation have reported that changes in m^6^A modification levels and the imbalance of regulatory factors are related to the activation and inhibition of cancer-related signalling pathways. Therefore, m^6^A modification is widely involved in the occurrence (Uddin et al., 2021), progression (Wang et al., 2020a), and drug resistance of cancer (Huang et al., 2020a) and may be a promising biomarker and potential therapeutic target for the diagnosis and prognosis of many kinds of tumours. High METTL3 (Vu et al., 2017), WTAP (Bansal et al., 2014; Naren et al., 2021), FTO (Li et al., 2017c), ALKBH5 (Shen et al., 2020a; Wang et al., 2020b), and YTHDF2 (Paris et al., 2019) expression has been observed in all subtypes of acute myelogenous leukaemia (AML), and high WTAP (Naren et al., 2021), ALKBH5 (Shen et al., 2020a; Wang et al., 2020b) and IGF2BP1 expression (Elcheva et al., 2020) are related to the poor prognosis of AML patients. The same phenomenon has been observed in solid tumours. METTL3, RBM15, KIAA1429, YTHDF1, YTHDF2, HNRNPA2B1, HNRNPC, and IGF2BP1/2/3 expression levels in lung cancer tissues are significantly higher than those in normal tissues (Shi et al., 2019; Zhang et al., 2020a; Li and Zhan, 2020; Sheng et al., 2020).

METTL3 may regulate the growth, differentiation, and apoptosis of AML cells by affecting the phosphoinositide 3-kinases (PI3K)/AKT pathway (Vu et al., 2017). Mechanistically, METTL3 promotes c-MYC, B-cell CLL/lymphoma 2 (BCL2), and phosphatase and tensin homologue (PTEN) mRNA translation by regulating m^6^A modification levels. Deleting METTL3 increases phosphorylated AKT (p-AKT) levels. METTL3 also regulates drug resistance and invasiveness of lung cancer cells by inducing m^6^A modification of enhancer of zeste homologue 2 (EZH2) mRNA in A549 cells (Chen et al., 2020a). In addition, it has been reported that the tumour suppressor miR-33a targets the 3′-UTR of METTL3 mRNA to reduce METTL3 expression, thus inhibiting A549 and NCI-H460 cell proliferation (Du et al., 2017). This suggests that METTL3 may be a new target for lung cancer therapy. Recently, Yankova et al. found that STM2457, a small molecule METTL3 inhibitor, reduced AML growth and increased apoptosis by reducing the expression of an mRNA known to cause leukaemia. Further animal experiments showed that STM2457 prolongs the survival time of various AML mouse models (Yankova et al., 2021). METTL14 acts in various solid tumours and leukaemia through different mechanisms. METTL14 expression is downregulated in AML cells. However, it still plays a carcinogenic role in AML. METTL14 increases MYB/MYC expression through the SPI1-METTL14-MYB/MYC signal axis to promote AML occurrence (Weng et al., 2018). METTL14 inhibits the migration and invasion of renal cancer cells by downregulating purinergic receptor P2X 6 (P2RX6) protein translation and ATP-P2RX6-Ca^2+^-p-ERK_1/2_-MMP9 signalling in renal cell carcinomas (Wang et al., 2019b).

The RNA demethylases FTO and ALKBH5 are also crucial in tumours. FTO may act as a tumour promoter. FTO increases the expression of myeloid zinc finger 1 (MZF1) by reducing m^6^A mRNA modification, and promotes lung cancer progression (Liu et al., 2018a). Knockdown of FTO increases the expression of tumour suppressor genes ASB2 and retinoic acid receptor alpha (RARA) and inhibits AML proliferation and differentiation (Li et al., 2017c). It also reduces the mRNA stability of ubiquitin-specific protease (USP7) and inhibits cancer cell growth (Li et al., 2019b).

In addition, some studies have focused on the function of m^6^A-binding proteins in tumours. YTHDF1 and YTHDF2 can be used as oncogenes and tumour suppressors. YTHDF1 deficiency regulates the transformation efficiency of cyclin-dependent kinase 2 (CDK2), cyclin-dependent kinase 4 (CDK4), and cyclin D1 (CCND1) through the Keap1-Nrf2-AKR1C1 pathway to inhibit tumour cell proliferation and xenograft tumorigenesis. YTHDF1 deletion also inhibits new lung adenocarcinoma (ADC) progression (Shi et al., 2019). However, the study also found that YTHDF1 knockdown leads to cell resistance to cisplatin, whereas high YTHDF1 expression leads to better clinical outcomes (Shi et al., 2019). The results of studies on the role of YTHDF2 in lung cancer are complex. One study reported that YTHDF2 promotes METTL3-induced tumorigenesis by increasing suppressor of cytokine signalling 2 (SOCS2) degradation (Chen et al., 2018). However, another study found that YTHDF2 overexpression inhibits non-small cell lung cancer (NSCLC) cell growth and invasion by promoting a decrease in yes-associated protein (YAP) mRNA in NSCLC cells (Jin et al., 2020). However, these studies have repeatedly confirmed the dual role of YTHDF1/2 in tumorigenesis and progression. IGF2BP1 exerts its carcinogenic function by regulating the expression of key transcriptional and metabolic factors, such as TNF receptor 2 (TNFR2), MYB, and MYC (Li et al., 2018a; Paris et al., 2019; Elcheva et al., 2020).

At present, m^6^A modification and its regulatory factors have proven to be crucial in the occurrence, metastasis, immune escape, and drug resistance of various tumours, including haematological tumours (Vu et al., 2017), respiratory tumours [lung cancer (Du et al., 2018) and nasopharyngeal carcinoma (Zheng et al., 2019)], digestive tract tumours (gastric cancer (Yang et al., 2020a), colorectal cancer (Ni et al., 2019; Shen et al., 2020b; Chen et al., 2021c), pancreatic cancer (Geng et al., 2020), and hepatocellular carcinoma (Chen and Wong, 2020)), urinary tumours [bladder cancer (Han et al., 2019), renal cell carcinoma (Zhuang et al., 2019), and prostate cancer (Zhu et al., 2021a)], reproductive system tumours [breast cancer (Cai et al., 2018), cervical squamous cell carcinoma (Wang et al., 2020c), epithelial ovarian cancer (Hua et al., 2018), and endometrial cancer (Liu et al., 2018b)], skin tumours [melanoma (Yang et al., 2019a; Jia et al., 2019), skin squamous cell carcinoma (Zhou et al., 2019)], and glioblastoma (Cui et al., 2017). Current research results show that m^6^A regulators may play a dual role in the pathogenesis of tumours, not only as oncogenes but as tumour suppressors. The biological effects of the same m^6^A regulator are different in different tumours. Some studies have reported the opposite role for an m^6^A regulator in the same cancer. In short, m^6^A modification can be used as a marker for a variety of tumours to diagnose and evaluate prognosis and potential therapeutic targets. However, our understanding of the role of m^6^A modification in tumours is still in its infancy. Numerous studies are still needed to explore the exact molecular mechanism of m^6^A and tumours to develop new targeted drugs for clinical treatment.

### 4.2 Diabetes Mellitus

m^6^A plays an important role in the pathogenesis of type 2 diabetes mellitus (T2D). It has been reported that the mRNA expression of RNA demethylase FTO in T2D patients is upregulated compared with that in a normal control group, inducing the increased expression of key genes involved in glucose and fat metabolisms, such as FOXO1, FASN, G6PC, and DGAT2. This suggests that FTO participates in glucose metabolism by regulating target gene expression (Yang et al., 2019b). In addition, some studies have found that METTL3/14 expression in the β cells of T2D patients and diabetic mice is decreased, leading to decreased β cell proliferation and impaired insulin secretion by reducing the m^6^A modification levels of several transcripts related to cell cycle progression, insulin secretion, and insulin/IGF1-AKT-PDX1 pathway (De Jesus et al., 2019; Wang et al., 2020d). In addition, loss of METTL3/14 is associated with abnormal glucose tolerance, hyperglycaemia, and hypoinsulinemia in neonatal mice (Liu et al., 2019; Men et al., 2019; Wang et al., 2020d). A recent study found that METTL3 mRNA and miR-25-3p expression were downregulated in PBMCs and retinal pigment epithelial (RPE) cells stimulated by high glucose. RPE cells overexpressing METTL3 could upregulate p-AKT levels through the miR-25-3p/PTEN axis, thus rescuing the viability of RPE cells stimulated by high glucose (Zha et al., 2020). However, inconsistently, Yang et al. found that METTL3 expression was upregulated in human diabetic cataract tissue samples and high glucose-induced human lens epithelial cells (HLECs), and the total level of m^6^A modification increased (Yang et al., 2020b). In summary, m^6^A modification is involved in the occurrence of T2D and its related complications. It is expected to provide a new diagnostic and treatment strategy for T2D and its complications.

### 4.3 Neurodegenerative Diseases

Currently, m^6^A modification is considered very important for nervous system development (Hess et al., 2013; Lence et al., 2016; Li et al., 2017a). In addition, some studies have found that abnormal m^6^A modifications are related to degenerative changes in the nervous system. Neurodegenerative diseases, including AD and PD, are caused by the gradual loss of neuronal structure or function. It has been reported that m^6^A modification levels are downregulated in 6-hydroxydopamine (6-OHDA)-treated PC12 cells and rat striatum, whereas 6-OHDA increases the level of oxidative stress and Ca^2+^ influx by inducing N-methyl-d-aspartate (NMDA) receptor one expression, leading to the death of dopaminergic neurons that eventually develops into PD (Chen et al., 2019b). In addition, some studies have focused on the correlation between m^6^A modification and AD. Compared with the control group, METTL3 expression in the cerebral cortex and hippocampus of AD model mice was upregulated, FTO expression was downregulated, and modification levels were significantly increased, suggesting that m^6^A methylation promotes AD development (Han et al., 2020). Mechanistic studies have reported that FTO activates the TSC1-mTOR-Tau signalling pathway by reducing m^6^A modification levels and then participates in the occurrence of AD (Li et al., 2018b; Annapoorna et al., 2019; Chen et al., 2019b). However, FTO expression was increased in the brains of ternary transgenic AD mice, and conditional knockout of FTO in the neurons of AD mice improved their cognitive ability (Li et al., 2018b). Previous studies have reported that FTO is associated with structural brain atrophy in healthy elderly subjects (Ho et al., 2010), and a prospective cohort study also found that FTO interacts with apolipoprotein E (APOE) to increase the risk of dementia, especially AD (Keller et al., 2011). In summary, the above studies showed that m^6^A modification is related to neurodegenerative changes, and its regulatory factors may be used as candidate therapeutic targets for neurodegenerative diseases. However, its role and mechanism need further exploration.

### 4.4 CVDs

Age is an independent risk factor for CVDs. Studies have shown that m^6^A modification may affect the occurrence and development of various CVDs. The level of m^6^A RNA methylation in pericytes of spontaneously hypertensive rats was decreased, suggesting that m^6^A is involved in blood pressure regulation (Wu et al., 2019a). In addition, under pressure overload stimulation, METTL3 induces compensatory cardiac hypertrophy by regulating the m^6^A modification of kinase and intracellular signal pathway transcripts. However, mice with conditional knockout of the METTL3 gene show the morphology and function of heart failure after stress or ageing stimulation (Dorn et al., 2019). Another study found that FTO expression increased after adipose factor-induced cardiomyocyte hypertrophy, whereas FTO knockout inhibited the hypertrophy of neonatal rat cardiomyocytes (Gan et al., 2013). Berulava et al. (2020) further confirmed these results. They found that the ejection fraction was significantly decreased in cardiomyocyte-specific knockout FTO mice, and heart failure progressed faster (Gan et al., 2013). However, another study found that increasing FTO expression in the hearts of mice with heart failure prevented the myocardial contractile transcript from degrading by reducing its m^6^A modification then reducing the decrease in myocardial contractility caused by ischaemia (Mathiyalagan et al., 2019). These studies suggest that m^6^A modification and its regulatory factors are crucial in maintaining normal myocardial homeostasis, compensatory myocardial hypertrophy, and heart failure progression.

In addition, m^6^A also acts in atherosclerosis progression. METTL14 increases the expression of mature miR-19a by upregulating the m^6^A modification of miR-19a and accelerates the proliferation of cardiovascular endothelial cells (Zhang et al., 2020b). Additionally, a study reported that METTL14 mediates endothelial cell inflammation, interacts with FOXO1, and promotes vascular cell adhesion molecule 1 (VCAM-1) and intercellular adhesion molecule 1 (ICAM-1) transcription, while METTL14 knockout inhibits the progression of atherosclerotic plaques in mice (Jian et al., 2020). It is believed that m^6^A modification affects the process of atherosclerosis by regulating cardiovascular endothelial proliferation and endothelial cell inflammation.

In summary, numerous studies have confirmed the correlation between m^6^A modification and CVDs, but further research needs to verify its established molecular changes and pathological process. In addition, most of the current reports focus on METTL3 and FTO, and the role of other m^6^A regulators, such as m^6^A binding proteins in CVDs, is still unclear. m^6^A modification still needs further exploration to provide a new treatment strategy for CVDs.

## 5 Conclusion and Perspectives

Alterations in the epigenetic transcriptome are key regulators of gene expression and cellular physiology. m^6^A, the most abundant internal modification of mRNAs and lncRNAs, is widely involved in regulating various cellular processes. Therefore, exploring the changes and molecular mechanisms of m^6^A modification in a pathological state and developing new targeted drugs will provide a new strategy for the early diagnosis and accurate treatment of diseases in the future.

Although several studies have reported on the functional role of m^6^A RNA methylation in ageing and related diseases, many major knowledge gaps remain to be filled. First, numerous studies have confirmed the correlation between m^6^A and age-related diseases. However, current research results are controversial. In tumours, for example, the same m^6^A regulatory factor may play different roles in different tumour types. For instance, METTL14 promotes the migration and invasion of breast cancer (Yi et al., 2020), whereas METTL14 downregulates the cancer-causing long-chain non-coding RNA X-inactive specific transcript (lncRNA XIST) and inhibits tumour proliferation and metastasis in colon cancer (Yang et al., 2020c). This may be due to the difference in disease types, but research on m^6^A is still in its infancy. The level of m^6^A modification, the biological role of regulatory factors in the occurrence and development of various diseases, and their molecular mechanisms require further study. There is still a way to go before m^6^A related drugs can be applied. Second, the epigenetic clock based on the DNA methylation site is recognised as the most promising marker of ageing and has been used to evaluate anti-ageing efficacy. m^6^A, a methylated form of epigenetics and DNA methylation, has been shown to function in ageing and ageing-related diseases. Whether it cooperates with DNA methylation to regulate gene expression during ageing or whether it has a potential relationship with other types of RNA modification or epigenetic methods remains to be further studied.

In addition, several reports have shown that m^6^A modification has great potential as a diagnostic marker and therapeutic target in the treatment of anti-ageing and age-related diseases, but few have identified inhibitors specifically targeting m^6^A regulatory proteins. Previous studies have found that the natural product rhein competitively binds the FTO active site *in vitro* (Chen et al., 2012), inhibits inflammation (Hu et al., 2019) and improves virus-induced lung injury (Shen et al., 2019). However, it is unclear whether m^6^A methylation regulation mediates these effects. Therefore, more drugs modified by m^6^A are required to fill this gap. In addition, the exact function of each m^6^A regulatory factor is not consistent in different cells, diseases, and even different stages of disease development. Our understanding of this is not comprehensive, which is also a challenge for applying m^6^A in anti-ageing therapy.

## Glossary

6-OHDA: 6-hydroxydopamine
AD: Alzheimer's disease
ADC: adenocarcinoma
AKT: Protein kinase B
ALKBH3: AlkB homologue 3
ALKBH5: AlkB homologue 5
AML: acute myeloid leukaemia
AMPK: AMP-activated protein kinase
ANGPTL4: angiopoietin-like 4
APOE: apolipoprotein E
ASB2: ankyrin repeat and SOCS box containing 2
ATG13: autophagy-related 13
BA9: Brodmann area 9
BCL2: B-cell CLL/lymphoma 2
CAMKK2: calcium/calmodulin-dependent protein kinase kinase 2
CCND1: cyclin D1
CDK2: cyclin-dependent kinase 2
CDK4: cyclin-dependent kinase 4
circNSUN2: circRNA NOP2/SUN domain family, member 2
circRNA: cyclic RNA
CRP: C-reactive protein
CVD: cardiovascular disease
DDR: DNA damage response
DGCR8: DiGeorge syndrome critical region 8
DNMT3A: DNA methyltransferase 3α
EGFR: epidermal growth factor
EZH2: enhancer of zeste homologue 2
FIP200: family interacting protein of 200kDa
FOXO3: Forkhead box O3
FTO: fat mass and obesity-related proteins
HDF: human diploid fibroblasts
HGPS: Hutchinson-Gilford progeria
HLEC: human lens epithelial cell
hMSC: human bone marrow mesenchymal stem cell
HNRNPC: heterogeneous nuclear ribonucleoprotein C
HNRNPG: heterogeneous nuclear ribonucleoprotein G
HNRNPA2B1: heterogeneous nuclear ribonucleoprotein A2B1
ICAM-1: intercellular adhesion molecule 1
IFN: interferon
IGF2BP: insulin-like growth factor 2 binding protein
IL: Interleukin
LPS: Lipopolysaccharides
lncRNA: long non-coding RNA;
lncRNA XIST: long-chain non-coding RNA X-inactive specific transcript
m6A: N6-methyladenosine; MAPK: mitogen-activated protein kinase
MERIP-seq: m6A methylation RNA immunoprecipitation sequencing
METTL3: RNA methyltransferase-like protein 3
METTL5: RNA methyltransferase-like protein 5
METTL14: RNA methyltransferase-like protein 14
METTL16: RNA methyltransferase-like protein 16
miRNA: microRNA
MK2: MAPKAPK2
mTOR: mammalian target of rapamycin
MZF1: myeloid zinc finger 1
NER: nucleotide excision repair
NF-κB: nuclear factor-κB
NHEJ: non-homologous end junction
NMDA: N-methyl-D-aspartate
NSCLC: non-small cell lung cancer
P13K: phosphoinositide 3-kinases
P2RX6: purinergic receptor P2X 6
p-AKT: phosphorylated AKT
PBMC: peripheral blood mononuclear cell
PD: Parkinson's disease
PGC1α: peroxisome proliferator-activated receptor gamma coactivator-1 alpha
PPARβ/δ: peroxisome proliferator-activated receptor
PTEN: phosphatase and tensin homologue
RARA: retinoic acid receptor alpha
RBM15: RNA -binding motif protein 15
RNA-seq: RNA sequencing
ROS: reactive oxygen species
RPE: retinal pigment epithelial
rRNA: ribosomal RNA
RUNX1: RUNT-related transcription factor 1
SA-βGAL: senescence-related β-galactosidase
SAM: S-Adenosyl Methionine
SASP: senescence-associated secretory phenotype
SFN: ammonium trifluoride
snoRNA: small nucleolar molecule RNA
snRNA: small nuclear RNA
SOCS2: suppressor of cytokine signalling 2
SRSF10: serine- and arginine-rich splicing factor 10
SRSF3: serine- and arginine-rich splicing factor 3
T2D: type 2 diabetes mellitus
TAZ: PDZ binding motif-based transcriptional coactivator
TLR: toll-like receptors
TNF: tumour necrosis factor
TNFR2: tumour necrosis factor receptor 2
tRNA: transfer RNA
ULK1: Unc-51 like autophagy activating kinase 1
ULK1/2: UNC-51-like kinase
USP7: ubiquitin specific protease 7
UV: ultraviolet
VCAM-1: vascular cell adhesion molecule 1
VIRMA: Vir-like m6A RNA methyltransferase associated protein
WS: Werner syndrome
WTAP: Wilms’ tumour 1-associating protein
XIST: X-inactive specific transcript
YAP: yes associated protein
YTHDC1: YTH domain containing 1
YTHDC2: YTH domain containing 2
YTHDF1: YTH domain family protein 1
YTHDF2: YTH domain family protein 2
YTHDF3: YTH domain family protein 3
ZCCH4: zinc finger CCHC-type containing 4
ZC3H13: zinc finger CCCH domain-containing protein 13
